# Supplementary material for: Load transfer mechanism and critical length of anchorage zone for anchor bolt
Source: PLoS One. 2020 Jan 17;15(1):e0227539. doi: 10.1371/journal.pone.0227539 (PMC6968847; doi:10.1371/journal.pone.0227539)
Supplement: S1 Table — (DOC) [file pone.0227539.s002.doc]

**Table 1. Parameters of mechanical properties of the test materials.**

| Anchor bolt | Types of anchor bolts | Diameter/ mm | Length/ mm | Tensile strength/ MPa | Yield strength/ MPa | Breaking force / kN |
| --- | --- | --- | --- | --- | --- | --- |
|  | Threaded steel | 20 | 2000 | 570 | 400 | 218.7 |
| Anchoring agent | Type | Characteristic | Length/ mm | Diameter/ mm | Gelation time/ s | Waiting time for installation / s |
|  | Z2350 | Intermediate speed | 500 | 23 | 91~180 | 480 |
