# Supplementary material for: Load transfer mechanism and critical length of anchorage zone for anchor bolt
Source: PLoS One. 2020 Jan 17;15(1):e0227539. doi: 10.1371/journal.pone.0227539 (PMC6968847; doi:10.1371/journal.pone.0227539)
Supplement: S2 Table — (DOC) [file pone.0227539.s003.doc]

Table 2. Mechanical parameters of materials.

| Performance parameters | Tensile strength /MPa | Yield strength/MPa | Shear modulus/GPa | Bulk modulus/GPa | Cohesion /MPa | Internal friction angle /° |
| --- | --- | --- | --- | --- | --- | --- |
| Anchoring agent | 15 | - | - | - | - | - |
| Anchor bolt | 570 | 400 | - | - | 12 | 32 |
| Surrounding rocks | 2.1 | 0.96 | 3.3 | 5.1 | 4.6 | 38 |
