# Supplementary material for: Load transfer mechanism and critical length of anchorage zone for anchor bolt
Source: PLoS One. 2020 Jan 17;15(1):e0227539. doi: 10.1371/journal.pone.0227539 (PMC6968847; doi:10.1371/journal.pone.0227539)
Supplement: S3 Table — (DOC) [file pone.0227539.s004.doc]

**Table 3. A comparison between the peak axial force and βL_b_ eigenvalues.**

| *P*_max_ | 0.9 | 0.91 | 0.92 | 0.93 | 0.94 | 0.95 | 0.96 | 0.97 | 0.98 | 0.99 | 0.995 |
| --- | --- | --- | --- | --- | --- | --- | --- | --- | --- | --- | --- |
| *βL*_b_ | 1.48 | 1.53 | 1.59 | 1.66 | 1.74 | 1.84 | 1.95 | 2.1 | 2.3 | 2.65 | 3 |
| *P*_max_ increment / ×10^-5^ | 189 | 173 | 155 | 136 | 117 | 97 | 79 | 59 | 40 | 20 | 10 |
