# Supplementary material for: Load transfer mechanism and critical length of anchorage zone for anchor bolt
Source: PLoS One. 2020 Jan 17;15(1):e0227539. doi: 10.1371/journal.pone.0227539 (PMC6968847; doi:10.1371/journal.pone.0227539)
Supplement: Attachment — Submitted filename: PONE-D-19-21977_reviewer response.pdf [file pone.0227539.s005.pdf]

# PLOS ONE

## Load transfer mechanism and critical length of anchorage zone for anchor bolt

--Manuscript Draft--

| | |
|---|---|
| **Manuscript Number:** | PONE-D-19-21977 |
| **Article Type:** | Research Article |
| **Full Title:** | Load transfer mechanism and critical length of anchorage zone for anchor bolt |
| **Short Title:** | Load transfer mechanism and critical length of anchorage zone for anchor bolt |
| **Corresponding Author:** | Suchuan Tian, Ph.D.<br>China University of Mining and Technology<br>Xuzhou, CHINA |
| **Keywords:** | anchorage zone, load transfer, pull-out test, critical length. |
| **Abstract:** | The length of anchorage zone of an anchor bolt affects the distribution of axial force and shear stress therein. Based on a shear–displacement model, the load distribution of anchor bolts in the elastic deformation stage was analysed. Moreover, the mechanical response of threaded steel anchor bolts with different anchorage lengths was explored through pull-out test and numerical simulation. The results showed that axial force and shear stress were negatively exponentially distributed within the anchorage zone of anchor bolts in which there were the maximum axial force and shear stress at the beginning of the anchorage zone. In the elastic deformation stage of the anchorage, the longer the anchorage length, the more uniformly the shear stress was distributed within the anchorage zone and the larger the ultimate shear stress; however, there was a critical anchorage length, which, when exceeded, the ultimate shear stress remained unchanged. The calculation formula for the critical anchorage length was deduced and a reasonable anchorage length determined. The research result provides an important theoretical basis for rapid design of support parameters for anchor bolts. |
| **Order of Authors:** | Xingliang Xu |
| | Suchuan Tian, Ph.D. |
| **Additional Information:** | |
| **Question** | **Response** |
| **Financial Disclosure**<br><br>Enter a financial disclosure statement that describes the sources of funding for the work included in this submission. Review the submission guidelines for detailed requirements. View published research articles from *PLOS ONE* for specific examples.<br><br>This statement is required for submission and **will appear in the published article** if the submission is accepted. Please make sure it is accurate. | This work was supported, and financed, by the General Program of the National Natural Science Foundation of China (51864044). |

Powered by Editorial Manager® and ProduXion Manager® from Aries Systems Corporation

| | |
|---|---|
| **Unfunded studies**<br>Enter: *The author(s) received no specific funding for this work.*<br><br>**Funded studies**<br>Enter a statement with the following details:<br>• Initials of the authors who received each award<br>• Grant numbers awarded to each author<br>• The full name of each funder<br>• URL of each funder website<br>• Did the sponsors or funders play any role in the study design, data collection and analysis, decision to publish, or preparation of the manuscript?<br>• **NO** - Include this sentence at the end of your statement: *The funders had no role in study design, data collection and analysis, decision to publish, or preparation of the manuscript.*<br>• **YES** - Specify the role(s) played.<br><br>* typeset | |
| **Competing Interests**<br><br>Use the instructions below to enter a competing interest statement for this submission. On behalf of all authors, disclose any competing interests that could be perceived to bias this work—acknowledging all financial support and any other relevant financial or non-financial competing interests.<br><br>This statement **will appear in the published article** if the submission is accepted. Please make sure it is accurate. View published research articles from *PLOS ONE* for specific examples. | The authors wish to confirm that there are no known conflicts of interest associated with this publication and there has been no significant financial support for this work that could have influenced its outcome. |

Powered by Editorial Manager® and ProduXion Manager® from Aries Systems Corporation

| | |
|---|---|
| **NO authors have competing interests**<br><br>Enter: *The authors have declared that no competing interests exist.*<br><br>**Authors with competing interests**<br><br>Enter competing interest details beginning with this statement:<br><br>*I have read the journal's policy and the authors of this manuscript have the following competing interests: [insert competing interests here]*<br><br>* typeset | |
| **Ethics Statement**<br><br>Enter an ethics statement for this submission. This statement is required if the study involved:<br><br>• Human participants<br>• Human specimens or tissue<br>• Vertebrate animals or cephalopods<br>• Vertebrate embryos or tissues<br>• Field research<br><br>Write "N/A" if the submission does not require an ethics statement.<br><br>General guidance is provided below. Consult the submission guidelines for detailed instructions. **Make sure that all information entered here is included in the Methods section of the manuscript.** | N/A |

Powered by Editorial Manager® and ProduXion Manager® from Aries Systems Corporation

| | |
|---|---|
| **Format for specific study types**<br><br>**Human Subject Research (involving human participants and/or tissue)**<br>• Give the name of the institutional review board or ethics committee that approved the study<br>• Include the approval number and/or a statement indicating approval of this research<br>• Indicate the form of consent obtained (written/oral) or the reason that consent was not obtained (e.g. the data were analyzed anonymously)<br><br>**Animal Research (involving vertebrate animals, embryos or tissues)**<br>• Provide the name of the Institutional Animal Care and Use Committee (IACUC) or other relevant ethics board that reviewed the study protocol, and indicate whether they approved this research or granted a formal waiver of ethical approval<br>• Include an approval number if one was obtained<br>• If the study involved *non-human primates*, add *additional details* about animal welfare and steps taken to ameliorate suffering<br>• If anesthesia, euthanasia, or any kind of animal sacrifice is part of the study, include briefly which substances and/or methods were applied<br><br>**Field Research**<br><br>Include the following details if this study involves the collection of plant, animal, or other materials from a natural setting:<br>• Field permit number<br>• Name of the institution or relevant body that granted permission | |
| **Data Availability**<br><br>Authors are required to make all data underlying the findings described fully available, without restriction, and from the time of publication. PLOS allows rare exceptions to address legal and ethical concerns. See the PLOS Data Policy and FAQ for detailed information. | Yes - all data are fully available without restriction |

| | |
|---|---|
| A Data Availability Statement describing where the data can be found is required at submission. Your answers to this question constitute the Data Availability Statement and **will be published in the article**, if accepted.<br><br>**Important:** Stating 'data available on request from the author' is not sufficient. If your data are only available upon request, select 'No' for the first question and explain your exceptional situation in the text box.<br><br>Do the authors confirm that all data underlying the findings described in their manuscript are fully available without restriction? | |
| **Describe where the data may be found in full sentences. If you are copying our sample text, replace any instances of XXX with the appropriate details.**<br><br>• If the data are **held or will be held in a public repository**, include URLs, accession numbers or DOIs. If this information will only be available after acceptance, indicate this by ticking the box below. For example: *All XXX files are available from the XXX database (accession number(s) XXX, XXX.).*<br>• If the data are all contained **within the manuscript and/or Supporting Information files**, enter the following: *All relevant data are within the manuscript and its Supporting Information files.*<br>• If neither of these applies but you are able to provide **details of access elsewhere**, with or without limitations, please do so. For example:<br><br>*Data cannot be shared publicly because of [XXX]. Data are available from the XXX Institutional Data Access / Ethics Committee (contact via XXX) for researchers who meet the criteria for access to confidential data.*<br><br>*The data underlying the results presented in the study are available from (include the name of the third party* | All relevant data are within the manuscript and its Supporting Information files. |

| | |
|---|---|
| *and contact information or URL).*<br>• This text is appropriate if the data are owned by a third party and authors do not have permission to share the data.<br><br>* typeset | |
| Additional data availability information: | Tick here if your circumstances are not covered by the questions above and you need the journal's help to make your data available. |

Powered by Editorial Manager® and ProduXion Manager® from Aries Systems Corporation

Manuscript Click here to access/download;Manuscript;Manuscript.docx

# Load transfer mechanism and critical length of anchorage zone for anchor bolt

**Xingliang Xu [a,b],Suchuan Tian [a*]**

[a] *Key Laboratory of Deep Coal Resource Mining, Ministry of Education of China, China University of Mining and Technology, Xuzhou, Jiangsu 221116, China*

[b] *Mining Department, Xinjiang Institute of Engineering, Urumqi, Xinjiang Uygur Autonomous Region, 830023,China*

**Abstract**: The length of anchorage zone of an anchor bolt affects the distribution of axial force and shear stress therein. Based on a shear–displacement model, the load distribution of anchor bolts in the elastic deformation stage was analysed. Moreover, the mechanical response of threaded steel anchor bolts with different anchorage lengths was explored through pull-out test and numerical simulation. The results showed that axial force and shear stress were negatively exponentially distributed within the anchorage zone of anchor bolts in which there were the maximum axial force and shear stress at the beginning of the anchorage zone. In the elastic deformation stage of the anchorage, the longer the anchorage length, the more uniformly the shear stress was distributed within the anchorage zone and the larger the ultimate shear stress; however, there was a critical anchorage length, which, when exceeded, the ultimate shear stress remained unchanged. The calculation formula for the critical anchorage length was deduced and a reasonable anchorage length determined. The research result provides an important theoretical basis for rapid design of support parameters for anchor bolts.

**Keywords**: anchorage zone, load transfer, pull-out test, critical length.

## 1 Introduction

As a key parameter affecting the design of bolt supports, the length of anchorage zone influences the anchoring force and support effect of anchor bolts, however, a theoretical basis for such a design remains absent, resulting in unreasonable anchorage lengths, thus leading to anchor support failure or extra cost[1,2]. Therefore, it is a challenge to guarantee that anchorage lengths satisfy design requirements while saving cost and therefore it is necessary to explore the load transfer mechanism and reasonable anchorage length of anchor bolts.

The load transfer mechanism of anchor bolts is a research hot-spot. The shear stress on anchor surface in the pull-out process can be divided into three parts: cohesion, mechanical self-locking force, and friction force[3]. Many mechanical models have been proposed: the shear lag model for an anchoring system based on the condition of considering bonding conditions of different interfaces[4], the simple trilinear constitutive model that describe the shear slip of the bonding interface between the anchor cable and grouting body[5], the stick-slip relationship and the trilinear stick–slip model established through pull-out tests on anchor bolts[6,7], the

three-parameter and two-parameter combined-power models of the distribution of axial force within the anchorage zone[8], the hyperbolic function model of load transfer by using mathematical–mechanical methods[9]. Zhu(2009) derived a function describing the distribution of frictional resistance on anchor bolts in an elastic homogeneous rock mass[10]. By applying displacement–shear stress theory and finite element analysis (FEA), the shear stress in the anchorage zone is distributed following a Gaussian function along the anchorage length. Through various *in situ* and laboratory tests[11], the distribution characteristics of axial force within the anchorage zone was obtained[12]. Despite the aforementioned research, no consensus has been reached as to the stress distribution in the anchorage zone.

As for research on anchorage length, the failure behaviours of bonded anchorage bodies under a fixed anchorage length was explored [13,14], the bearing capacity did not significantly increase when the anchorage length exceeded the critical anchorage length[15]. Huang(2018) proposed a method for calculating the critical anchorage length of anchor bolts and verified its feasibility through engineering case studies[16]. Based on the bonding effect, The anchorage length has a serious influence on the bearing capacity of anchor bolts and shear stress on interfaces under the effect of cyclic load[17-19]. The calculation formula for the critical anchorage length of anchor bolts can be deduced according to the principle of displacement compatibility between the anchorage body and surrounding rock[20-22]. Liu(2010) thought that the anchorage length has to exceed 20 times the diameter of the anchor bolt when applying full-thread GFRP anchor bolts in situ[23]. The aforementioned research achievements remain mostly hypothetical, and do not take the design requirements of actual parameters of anchor bolts into account.

In the present study, the mechanical properties and stress distribution characteristics of the anchorage zone under different anchorage lengths were explored to reveal the load transfer mechanism of the anchorage zone and propose a method for designing a reasonable anchorage length of anchor bolts.

## 2 Analysis of mechanical properties of the anchorage zone

An anchoring system comprises: anchor bolts, anchoring agent, surrounding rocks, and parts of the anchor bolts. An anchor bolt is divided into exposed, free, and anchorage zones (Fig 1) along its length. When the anchor bolt is subjected to pull-out effects, the axial force in the free zone is transferred to the anchorage zone due to elastic deformation therein. Based on bonding, friction, and mechanical meshing between the anchor bolt and anchoring agent, the circular binding body formed by the anchoring agent, and the effect of the borehole wall,

load is transferred to the surrounding rock. The anchoring force refers to the binding force between the anchorage zone of anchor bolts and a rock mass, that is, the constraint force on the anchor bolt from the surrounding rock, which is frequently considered as an important index with which to measure anchor integrity.

Based on the force transfer process of anchoring system, it can be seen that there are three mechanical interfaces in the anchoring system. When analysing the mechanical properties of the anchorage zone in the elastic stage, the two interfaces (including anchor bolt–anchoring agent and anchoring agent–borehole wall interfaces) were explored. When applying pull-out force to an anchor bolt, the shear stress on the anchorage zone depends on the coupling mechanism between interfaces[24,25]. For grouted anchor bolts, relative displacement occurs between the anchor bolts and surrounding slurry, thus failing in slip on the anchor bolt–anchoring agent interface. Then, the shear stress on the interface is lower than the ultimate shear strength of the interface. For a resin anchor bolt, the anchor bolt is deformed with its anchoring agent, generally failing in slip on the anchoring agent–borehole wall interface. In this case, the shear stress on the interface is equivalent to the ultimate shear strength. The latter was explored in the present study.

According to different deformation forms of anchoring agent–borehole wall interface, the pull-out process of anchor bolts into three stages was simplified[5,26], as shown in Fig 2.

In Stage I (elastic deformation stage), the shear stress is proportional to the shear displacement of the interface which is intact. In this case, $0 \leq \mu \leq \mu_1$ and the relationship between shear stress $\tau$ and displacement $\mu$ is expressed as follows:

$$\tau = \frac{\tau_1}{\mu_1}\mu \qquad (1)$$

where, $\tau_1$ and $\mu_1$ refer to the ultimate bonding strength of anchorage body and shear displacement at the ultimate bonding strength of anchorage zone, respectively.

In Stage II (interface softening and damage stage), the interface is partly damaged and therefore shear stress linearly declines with shear displacement. In this context, $\mu_1 \leq \mu \leq \mu_2$ and the shear stress can be calculated as follows:

$$\tau = \frac{\tau_1 - \tau_2}{\mu_1 - \mu_2}\mu + \frac{\tau_2\mu_1 - \tau_1\mu_2}{\mu_1 - \mu_2} \qquad (2)$$

where, $\tau_2$ and $\mu_2$ are the residual bonding strength of anchorage zone and the minimum shear displacement under the residual bonding strength of the anchorage zone, respectively.

In Stage III (residual strength stage), the interface was completely damaged; in this context, $\mu \geq \mu_2$ and the

shear stress is expressed as follows:

$$\tau = \tau_2 \tag{3}$$

By modifying the micro-element model[27,28], the distribution equation for axial force in the anchorage zone is expressed as follows:

$$P(x) = \frac{e^{\beta x} - e^{\beta(2L_b - x)}}{(1 - e^{2\beta L_b})} P \tag{4}$$

The equation for shear stress distribution of anchoring agent–borehole wall interface is as follows:

$$\tau(x) = \frac{e^{\beta x} + e^{\beta(2L_b - x)}}{\pi D(e^{2\beta L_b} - 1)} \beta P \tag{5}$$

where, $D$, $P$, and $\beta$ separately denote the diameter of the borehole, pull-out force of an anchor bolt, and a material parameter given by:

$$\beta^2 = \frac{4\tau_1}{\mu_1 D E_a} \tag{6}$$

where, $E_a$ is the elastic modulus of the anchorage zone.

According to Equations 4 and 5, the distribution curves of axial force and shear stress in the anchorage zone are drawn, as shown in Fig 3.

The axial force and shear stress of anchorage body monotonically decreased from the beginning to the end of the anchorage zone while the rate of change thereof gradually declined. At the beginning ($x = 0$) of the anchorage zone, the axial force and shear stress on the anchorage body were at a maximum and the axial force was equivalent to that in the free zone of an anchor bolt. On condition of having sufficient pull-out force, relative displacement and damage first appeared at the beginning of the anchorage zone. Afterwards, damage gradually extended to the end of the anchorage zone. At the end ($x = L_b$) of the anchorage zone, the axial force was zero while there was still a residual shear stress present.

## 3 The influence of anchorage length on the stress distribution in the anchorage zone

Bolt support is complex and concealed from observers, so it is hard to measure the deformation and stress on the anchor bolts in field. It is necessary to verify the result obtained through theoretical analysis by conducting laboratory testing to analyse the load transfer characteristics of an anchoring system.

### 3.1 Test materials and platform

In the test, the left-handed threaded steel anchor bolts were applied and the thick-walled steel tube and resin

cartridge were separately taken as the anchoring matrix and binding material (Fig 4). Considering the binding effect of this resin anchoring agent, a seamless steel tube with the inner diameter of 30 mm was used, in which threads were processed. The parameters of test materials are shown in Table 1.

**Table 1. Parameters of mechanical properties of the test materials.**

| | | | | | | |
|---|---|---|---|---|---|---|
| Anchor bolt | Types of anchor bolts | Diameter/ mm | Length/ mm | Tensile strength/ MPa | Yield strength/ MPa | Breaking force / kN |
| | Threaded steel | 20 | 2000 | 570 | 400 | 218.7 |
| Anchoring agent | Type | Characteristic | Length/ mm | Diameter/ mm | Gelation time/ s | Waiting time for installation / s |
| | Z2350 | Intermediate speed | 500 | 23 | 91~180 | 480 |

The pull-out test was conducted by applying an LW-1000 horizontal tensile test machine (Fig 4). Before the test, the back collet was fixed by using a latch and the end of the anchor bolt with threads was placed into the back collet and fixed through pallet nuts. Moreover, the anchor end (seamless steel tube) was fixed using a front collet. During the test, the front collet was driven through a piston and a pull rod to move away from the back collet to simulate a pull-out force on the anchor bolt. A sensor was used to collect and transfer data (in real time) to a computer.

### 3.2 Test scheme

Strain gauges were distributed in the anchorage zone at 100 mm intervals to measure the stress and strain on the anchorage body under the pull-out effect and analyse the change in stress in the anchorage zone. TS3890 static resistance strain gauges were used to measure the strain (Fig 5).

During the test, the four-level loads (25, 50, 75, and 100 kN) were separately applied to the anchorage zones with the anchorage lengths of 500, 1000, and 1500 mm. The load was maintained for 3 s and the mechanical response of the anchorage body under different anchorage lengths and pull-out loads analysed.

### 3.3 The influence of anchorage length on stress distribution in the anchorage zone

#### 3.3.1 Shear stress

Based on measured parameters of anchor bolts for mining service and surrounding rocks, the elastic moduli of the anchorage body and resin cartridge, diameter of anchor bolt, diameter of borehole, and Poisson's ratio of surrounding rocks were 200 GPa, 3 GPa, 20 mm, 30 mm, and 0.24, respectively. On this basis, the curves for comparing changes of shear stress are shown in Fig 6.

Fig 6 shows the shear stress distributions on interfaces in the anchorage zone for anchorage lengths of 0.5, 1, 1.5, and 2 m when the pull-out force was 50 kN. It can be seen from the Fig 6 that under the same pull-out force and different anchorage lengths, the shear stress on the interfaces did not change linearly but reached a maximum

at the beginning of the anchorage zone and gradually reduced to zero with increasing distance from the beginning. The interface was mainly stressed close to the end of the free zone. The shorter the anchorage length, the more uniformly the shear stress was distributed along the anchorage zone and the higher the maximum shear stress on the interfaces. With increasing anchorage zone length, the shear stress on the interfaces decreased and was gradually transferred to the section near the end of the anchorage zone. At the end nearest the applied load (near end, hereinafter), debonding occurred and the shear stress was gradually transformed into a frictional resistance. In this case, the shear stress on the anchorage body was low at a certain distance from the near end. When the anchorage length reached a certain level, the distribution curves of shear stress on interfaces gradually coincided, implying that further increasing the anchorage length had little significant effect on the maximum shear stress.

A FLAC3D numerical model was established. During simulation, the anchorage interface in a rock mass was simulated by applying interface elements while contact elements were used to simulate the contact interface of media effecting force transfer. The interface elements were used for simulation based on the Mohr-Coulomb model. The contact constitutive model for elements was adjusted through parameter setting to simulate the true interface, in which anchor bolt was simulated by using an isotropic elasticity model.

**Table 2. Mechanical parameters of materials.**

| Performance parameters | Tensile strength /MPa | Yield strength/MPa | Shear modulus/GPa | Bulk modulus/GPa | Cohesion /MPa | Internal friction angle /° |
|---|---|---|---|---|---|---|
| Anchoring agent | 15 | - | - | - | - | - |
| Anchor bolt | 570 | 400 | - | - | 12 | 32 |
| Surrounding rocks | 2.1 | 0.96 | 3.3 | 5.1 | 4.6 | 38 |

The model measures 1.0 m × 1.0 m × 1.2 m (length × width × height) and the total length of anchor bolt was 1.2 m, including an anchorage zone and a free zone of 1.0 m and 0.2 m long, respectively. The anchor bolt, with a diameter of 20 mm, was aligned in the centre of the model, with a thickness of anchoring agent of 5 mm simulated. Fig 7 shows stress distributions in the anchoring agent at anchorage lengths of 0.5 and 1.0 m.

Shear stress was mainly distributed within a small zone in the near end and shear stress was exponentially distributed and gradually declined from the near end to the far end. The longer the anchorage, the wider the distribution of shear stress and the lower the corresponding shear stress; moreover, the longer the anchorage, the nearer the shear stress was to zero in the anchorage zone (it was even negative in places). The stress distribution on the anchorage body in the numerical model shows similarities with analytical solutions based on the shear–slip model. In engineering practice, it is necessary to reinforce the vicinity of the interface as much as possible to guarantee the strength of surrounding rocks near the interface and also ensure the integrity of anchorage in the initial segment.

3.3.2 Analysis of axial stress

The axial stress is given by:

$$\sigma_i = \varepsilon_i E_s \qquad (7)$$

where, $\sigma_i$ and $\varepsilon_i$ denote the axial force and strain at point $i$, respectively.

The axial force at the borehole mouth was equivalent to that in the free zone. With a resin anchoring agent, the axial force distribution varied and was different from the equivalent distribution in the free zone. The axial stress gradually decreased from the outer end to the tailing end of the anchor because the cohesion at the near end of the anchor bolt was gradually overcome with increasing pull-out load and the interface at the tailing end was constantly driven to resist the pull-out load. Additionally, the axial stress of anchor bolt correspondingly increased. The axial force on the anchor bolt within the anchorage zone also increased with the external load applied to the anchor bolt.

As shown in Fig 8, when applying a pull-out force of 50 kN, the axial force varied quasi-linearly when the anchorage length was 0.5 m. With increasing anchorage length, the axial force of anchor bolts became less uniform. When the anchorage length was 1500 mm, the axial force was mainly distributed in the vicinity of the borehole mouth and decreased with distance therefrom. At a certain anchorage length, the axial force tended to zero and the peak axial force was unaffected; however, due to the increase in anchorage length, the zone over which the axial force was distributed expanded and therefore the anchor bolt further from the anchorage interface was subjected to a small axial force. That is, it exhibited sufficient bearing capacity and can thus bear more load. The result obtained through numerical simulation was consistent with that obtained by analytic calculation.

3.4 The influence of pull-out force on the stress distribution in the anchorage zone

3.4.1 Distribution of axial stress under different pull-out forces

When the anchorage length was 1.0 m, the changes in axial stress of anchorage zone under three-level pull-out forces (25, 50, and 75 kN) were simulated. In Fig 9, the axial force is seen to be non-linearly distributed along the anchor. In the elastic stage, anchor bolts showed the same trend of stress distribution with increasing load, moreover, stress changes were mainly found at the beginning of the anchorage zone where the ultimate pull-out force was first mobilised. On this basis, it can be inferred that the anchorage body of an anchor bolt was first damaged at the beginning of its anchorage zone.

3.4.2 Distribution of shear stress under different pull-out forces

Under low load, the interface between the anchoring agent and the anchor bolt at the borehole mouth was subjected to elastic deformation. In this case, the anchorage body was undamaged and shear stress within the

anchorage zone gradually reduced and was uniformly distributed. With increasing load, the shear stress rapidly rose to its peak within a short distance from the borehole mouth: this implied that shear failure started to occur at the beginning of the anchorage zone and the failure gradually extended to the deeper anchorage interface with increasing load. As the maximum shear stress remained unchanged, the locus of the peak shear stress shifted to the deeper anchorage zone. With a large anchorage length, there was a wider response range to external load within the anchorage zone, so the anchorage body can bear a larger load, thus improving the bearing capacity of the anchorage zone. By analysing Fig 10, it can be found that, within the ultimate bearing range, the larger the pull-out force, the less uniform the stress distribution; the longer the anchorage, the more centralised the shear stress on the interface at the beginning of the anchorage zone.

## 4 Determination of reasonable anchorage length

### 4.1 Determination of critical anchorage length

It can be seen from Fig 6(d) and Fig 8(d) that there was a critical length of anchorage zone under the effect of pull-out force, beyond which the ultimate bearing capacity of the anchor bolts did not increase. When the external load reached a certain level, the anchorage layer changed from one undergoing elastic deformation to elasto-plastic deformation and the shear stress on the anchorage interface did not continue to increase. To guarantee anchorage body function, the maximum shear stress on the anchorage zone cannot exceed the ultimate shear strength of the anchorage body–rock interface, which was taken as the main controlling condition for determining the anchorage length. In this context, the resistance at the beginning of the anchorage zone was equivalent to the ultimate shear stress [$\tau$] on the interface. By simultaneously using Equation 4, the ultimate pull-out force of the anchorage zone can be obtained thus:

$$P_{\max} = \frac{\pi D[\tau](e^{2\beta L_b} - 1)}{\beta(1 + e^{2\beta L_b})} \qquad (8)$$

Owing to $\tanh x = \frac{e^x - e^{-x}}{e^x + e^{-x}}$, assuming $x=\beta L_b$, the following result can be attained:

$$P_{\max} = \frac{\pi D[\tau]}{\beta} \tanh(\beta L_b) \qquad (9)$$

The ultimate bearing capacity of anchoring system increased with increasing anchorage length and shear capacity of the anchorage interface. With the constant growth of anchorage length, the bearing capacity of the anchoring system increased, then stabilised, as shown in Fig 11.

When $\beta L_b$ was infinite, $\tanh(\beta L_b)$ tended to unity; however, in practical engineering, it not only needs to be

technically satisfactory, but also cost-effective. According to the peak, and incremental, axial force, the eigenvalues of the system can be attained (Table 3).

**Table 3. A comparison between the peak axial force and βLb eigenvalues.**

| $P_{max}$ | 0.9 | 0.91 | 0.92 | 0.93 | 0.94 | 0.95 | 0.96 | 0.97 | 0.98 | 0.99 | 0.995 |
|---|---|---|---|---|---|---|---|---|---|---|---|
| $\beta L_b$ | 1.48 | 1.53 | 1.59 | 1.66 | 1.74 | 1.84 | 1.95 | 2.1 | 2.3 | 2.65 | 3 |
| $P_{max}$ increment / $\times 10^{-5}$ | 189 | 173 | 155 | 136 | 117 | 97 | 79 | 59 | 40 | 20 | 10 |

According to the corresponding relationship between $P_{max}$ and $\beta L_b$ in Table 3, it can be seen that the increment of $\beta L_b$ increased with $P_{max}$. This meant that, after reaching a certain critical value, the anchorage length needs to be increased by much more when augmenting the axial force on the anchor bolt by the same amount. Therefore, there is a certain reasonable length range, in which technical and economic effects can both be satisfied. When $P_{max} > 0.9$, it is supposed that $k$ denotes the increment of $\beta L_b$ required for the same increase in axial force on the anchor bolt, that is, the efficiency of increasing the peak axial force of anchor bolt by increasing the anchorage length (Fig 12) can be deduced.

As shown in Fig 12, when $P_{max} < 0.98$, the increment in $\beta L_b$ and $k$ increased slightly; when $P_{max} \geq 0.98$, the increment in $\beta L_b$ and $k$ both increased, therefore, $P_{max} = 0.98$ can be considered as a criterion for discriminating a reasonable anchorage length, with which economic principles are also satisfied on the premise of realising the desired technical end. In this case, $\beta L_b = 2.3$, so the reasonable anchorage length of such anchor bolts was $0.435\beta$, that is, $0.87\sqrt{\frac{\tau_1}{\mu_1 D E_a}}$.

## 5 Conclusions

(1) Based on the shear–displacement model, the analytical expressions for the distribution of axial force on the anchorage body and shear stress on the anchorage body–surrounding rock interface along the anchorage zone were attained. Furthermore, based on the shear–displacement model, it was found that the axial force decreased in a non-uniform manner along the anchor bolt to the deeper anchorage zone. Moreover, the shear stress on interface at the beginning of anchorage zone of the anchor bolts was maximised, then decreased along anchor.

(2) The influence of anchorage length on the stress distribution along an anchor bolt was obtained: in the elastic deformation stage, the longer the anchorage length, the more uniform the shear stress distribution along the anchorage zone and the higher the maximum shear stress on the interface. Beyond a certain critical anchorage length, further increases therein caused no significant influence on the maximum shear stress.

(3) It was shown that there was a critical anchorage length: as the peak axial force on the anchor bolts

exhibited a hyperbolic tangent relationship with the anchorage length, it was determined that the technical and economic effects of an anchor bolt support system can be realised when the optimal anchorage length was 0.435$\beta$.

## Conflict of interest

The authors wish to confirm that there are no known conflicts of interest associated with this publication and there has been no significant financial support for this work that could have influenced its outcome.

## Acknowledgements

This work was supported, and financed, by the General Program of the National Natural Science Foundation of China (51864044). The authors would like to thank the Editor and the Reviewers for their helpful and constructive comments.

## References

**1.** Hsiao FY, Wang CL, Chern JC. Numerical simulation of rock deformation for support design in tunnel intersection area. Tunnelling and Underground Space Technology 2009; 24(1):14-21.

**2.** Kaiser PK, Cai M. Design of rock support system under rockburst condition. Journal of Rock Mechanics and Geotechnical Engineering 2012; 4(3):215-227.

**3.** Li C, Stillborg B. Analytical models for rock bolts. International Journal of Rock Mechanics and Mining Sciences 1999; 36(8):1013-1029.

**4.** Steen M, Valles JL. Interfacial bond conditions and tress distribution in a two- dimensionally reinforced brittle-matrix composite. Composites Science and Technology 1998; 58(3-4):313-330.

**5.** Benmokrane B, Chennouf A, Mitri HS. Laboratory evaluation of cement-based grouts and grouted rock anchors. International Journal of Rock Mechanics and Mining Sciences & Geomechanics Abstracts 1995; 32(7):633-642.

**6.** Nemcik J, Ma SQ, Aziz N, Ren T, Geng XY. Numerical modelling of failure propagation in fully grouted rock bolts subjected to tensile load. International Journal of Rock Mechanics and Mining Sciences 2014; 71:293-300.

**7.** Martín LB, Tijani M, Hadj-Hassen F. A new analytical solution to the mechanical behaviour of fully grouted rockbolts subjected to pull-out tests. Construction and Building Materials 2011; 25(2):749-755.

**8.** Zhu Y, Wei J, Liao CH. The combined-power model method on determining the bounding length of prestressed anchoring rope. Journal of Wuhan University of Technology 2005; 08:60-63.

**9.** Zhang JR, Tang BF. Hyperbolic function model to analyze load transfer mechanism on bolts. Chinese Journal of Geotechnical Engineering 2002; 24(2):188-192.

**10.** Zhu XG, Yang Q. Analyzing and studying factors for determining neutral point position of fully grouted rock bolt. Rock and Soil

Mechanics 2009; 11:3386-3392.

**11.** Jiang ZX. A Gauss curve model on shear stress along anchoring section of anchoring rope of extensional force type. Chinese Journal of Geotechnical Engineering 2001;23(6):659-662.

**12.** Štefaňák J, MičaL, Chalmovský J, Leiter A, Tichý P. Full-scale Testing of Ground Anchors in Neogene Clay. Procedia Engineering 2017; 17:1129-1136.

**13.** Ivanović A, Neilson RD. Modelling of debonding along the fixed anchor length. International Journal of Rock Mechanics and Mining Sciences 2009; 46(4):699-707.

**14.** Akisanya AR, Ivanović A. Debonding along the fixed anchor length of a ground anchorage. Engineering Structures 2014; 74:23-31.

**15.** Zeng XM， Lin DL, Li SM, Zuo K, Xu XH, Du NB. Comprehensive research of critical anchorage length problem of rod anchorage structure. Chinese Journal of Rock Mechanics and Engineering 2009; 28(S2):3609-3625.

**16.** Huang MH, Zhao MH, Chan CF. Influence of anchorage length on stress in bolt and its critical value calculation. Rock and Soil Mechanics 2018; 39(11):4033-4041+4062.

**17.** Kılıc A, Yasar E, Celik AG. Effect of grout properties on the pull-out load capacity of fully grouted rock bolt. Tunnelling and Underground Space Technology 2002; 17(4):355-362.

**18.** Tistel J, Grimstad G, Eiksund G. Testing and modeling of cyclically loaded rock anchors. Journal of Rock Mechanics and Geotechnical Engineering 2017; 9(6):1010-1030.

**19.** Oh BH, Kim SH. Realistic models for local bond stress-slip of reinforced concrete under repeated loading. Journal of Structural Engineering 2007;133(2):216-24.

**20.** Thenevin I, Blanco-Martín L, Hadj-HassenF, Schleifer J, Lubosik Z, Wrana A. Laboratory pull-out tests on fully grouted rock bolts and cable bolts: Results and lessons learned. Journal of Rock Mechanics and Geotechnical Engineering 2017; 9(5):843-855.

**21.** Long Z, Zhao MH, Zhang EX, Liu JL. A simplified method for calculating critical anchorage length of bolt. Rock and Soil Mechanics 2010;31(9):2991-2995.

**22.** Zhang J. Sahng YQ, Ye B. Analytical calculations of critical anchorage length of bolts. Chinese Journal of Rock Mechanics and Engineering 2005; 24(7):1134-1138.

**23.** Liu YH, Yuan Y. Experimental research on anchorage performance of full-thread gfrp bonding anchor bolts. Chinese Journal of Rock Mechanics and Engineering 2010; 29(02):394-400.

**24.** Ito F, Nakahara F, Kawano R, Kang S , Obara Y. Visualization of failure in a pull-out test of cable bolts using X-ray CT. Construction and Building Materials 2001; 15(5-6):263-270.

**25.** Assaad JJ, Gerges N. Styrene-butadiene rubber modified cementitious grouts for embedding anchors in humid environments. Tunnelling and Underground Space Technology 2019; 84:317-325.

**26.** You CA, Zhan YB, Liu QY, Sun LL, Wang KB. Analysis of interfacial slip mesomechanics in anchorage section of prestressed anchor cable. Chinese Journal of Rock Mechanics and Engineering 2009; 28(10):1976-1985.

**27.** Li HZ, Li XH. Determination of rational anchorage length of bolt based on slip-debonding failure mode of interface. Rock and Soil Mechanics 2017; 38(11):3106-3112.

**28.** Wang HT, Wang Q, Wang FQ, Li SC, Wang DC, Ren YX, Guo NB, Zhang SG. Mechanical effect analysis of bolts in roadway under different anchoring lengths and its application. Journal of China Coal Society, 2015; 40(3):509-515.

Figures Click here to access/download;Figure;Figures.docx

# Figures

Fig 1. Force transfer mechanism in an anchor bolt.

Fig 2. Shear stress–displacement relationship on the anchoring agent–borehole wall interface.

Fig 3. Distributions of axial force and shear stress in the anchorage zone.

Fig 4. Apparatus for the pull-out test and test materials.

Fig 5. Distribution of strain gauges.

Fig 6. Shear stress distribution of anchorage body under a same pull-out force and different anchorage lengths.

Fig 7. Stress distribution in the anchoring agent at different anchorage lengths.

Fig 8. Distributions of axial stress in the anchorage zone at different anchorage lengths and a given pull-out force.

Fig 9. The distribution of axial force in the anchorage zone under different pull-out forces and the same anchorage length.

Fig 10. Distributions of shear stress in the anchorage zone under different pull-out forces at the same anchorage length.

Fig 11. Relationships between peak, and incremental, axial force in the anchorage zone with anchorage length.

Fig 12. Relationships between peak, and incremental, axial forces in the anchorage zone with anchorage length.

**Fig 1. Force transfer mechanism in an anchor bolt.**

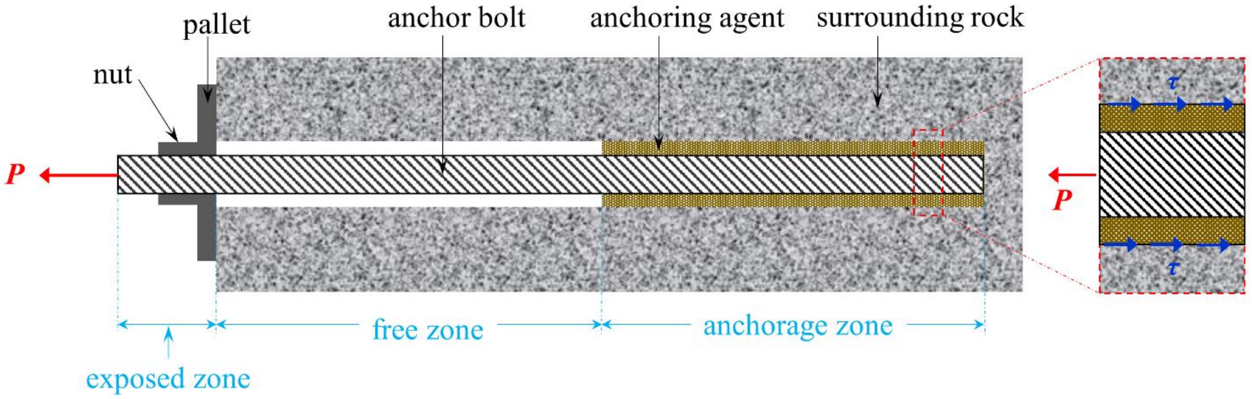

**Fig 2. Shear stress–displacement relationship on the anchoring agent–borehole wall interface.**

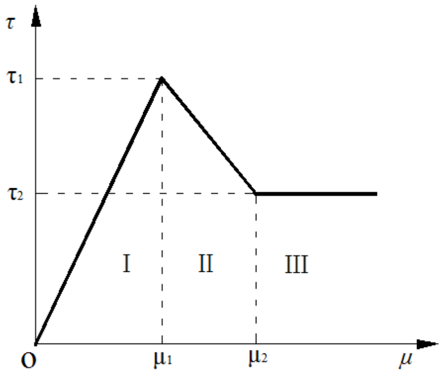

**Fig 3. Distributions of axial force and shear stress in the anchorage zone.**

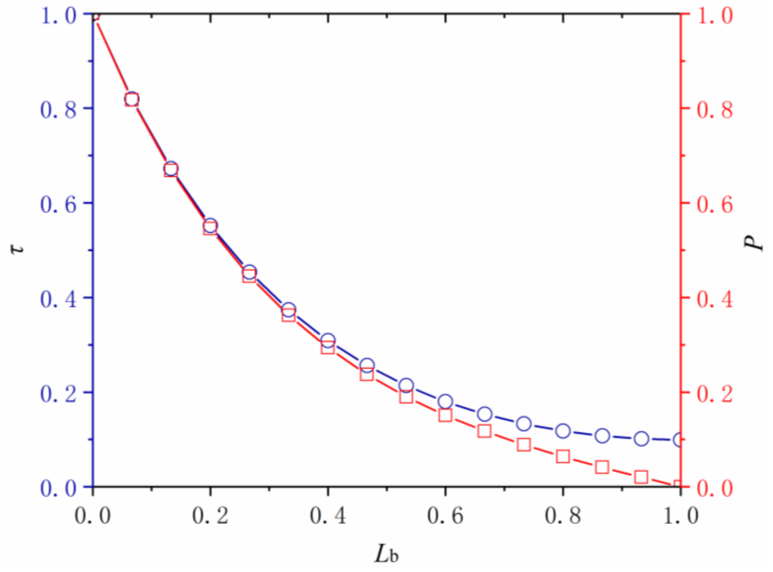

**Fig 4. Apparatus for the pull-out test and test materials.**

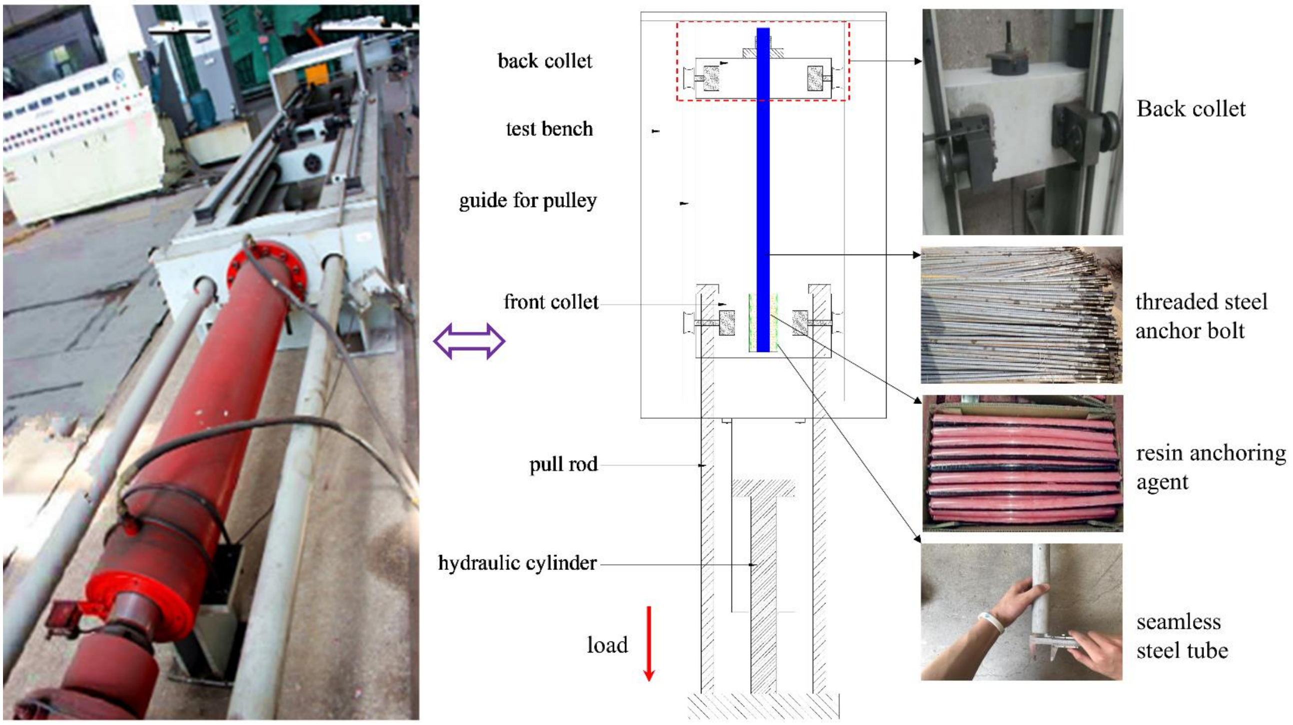

**Fig 5. Distribution of strain gauges.**

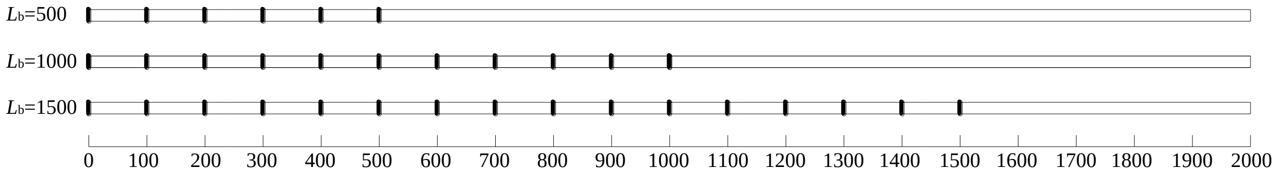

**Fig 6. Shear stress distribution of anchorage body under a same pull-out force and different anchorage lengths.** Anchorage lengths of 0.5 m (a), 1.0 m (b), and 1.5 m (c).

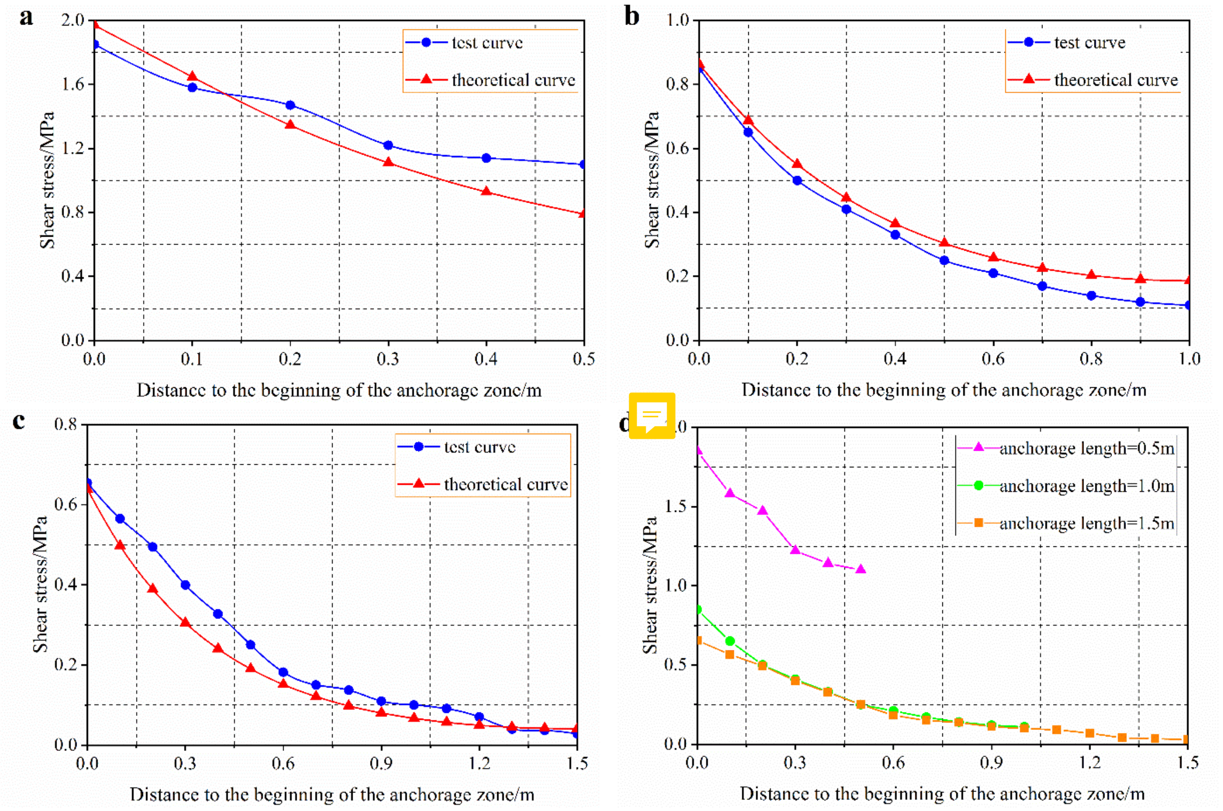

**Fig 7. Stress distribution in the anchoring agent at different anchorage lengths.** (a) 0.5 m; (b) 1.0 m

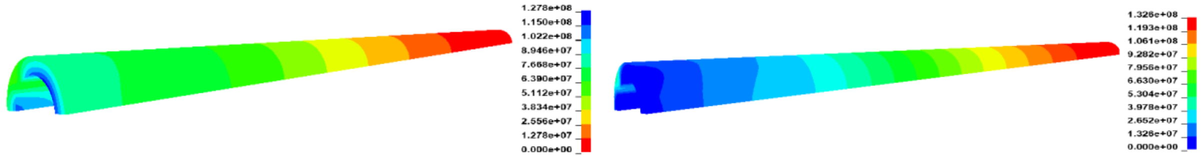

**Fig 8. Distributions of axial stress in the anchorage zone at different anchorage lengths and a given pull-out force.** Anchorage lengths of 0.5 m (a), 1.0 m (b), and 1.5 m (c), (d) is test curves of three length.

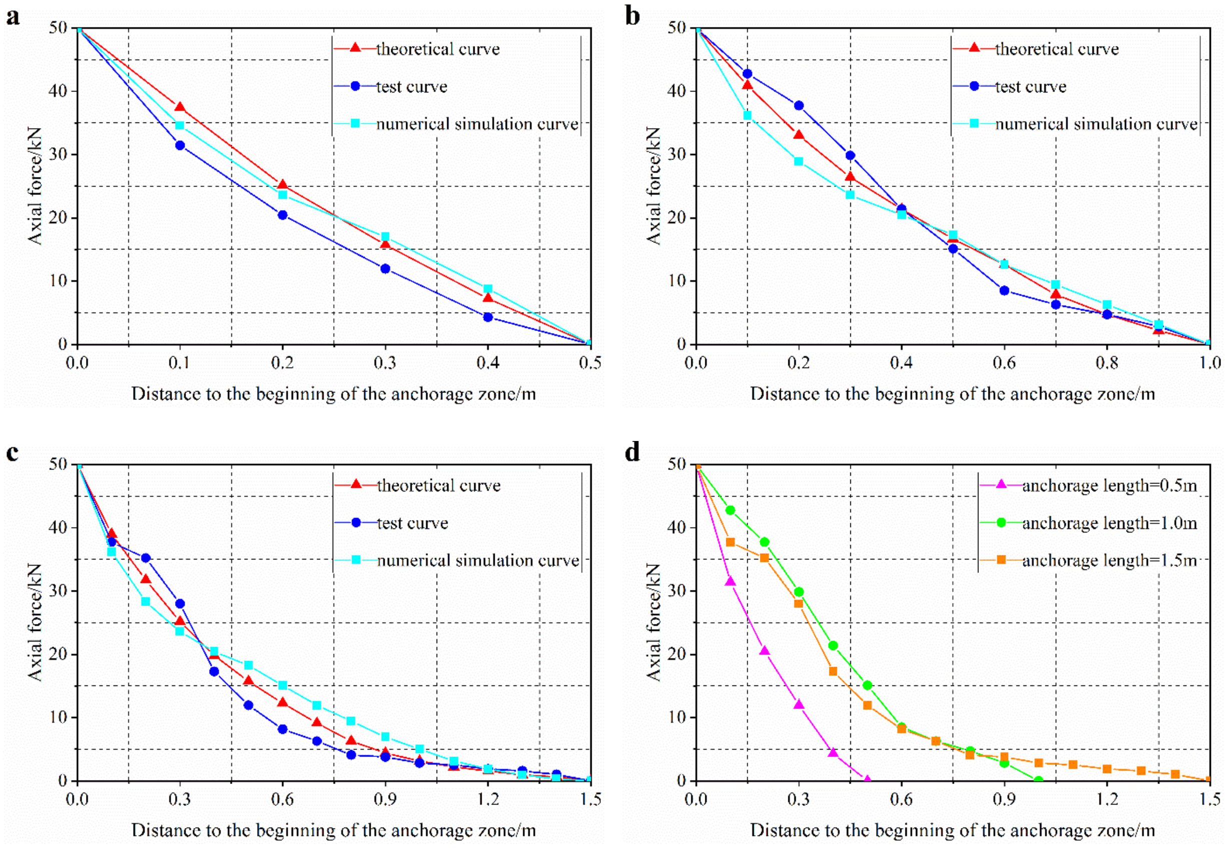

**Fig 9. The distribution of axial force in the anchorage zone under different pull-out forces and the same anchorage length.**

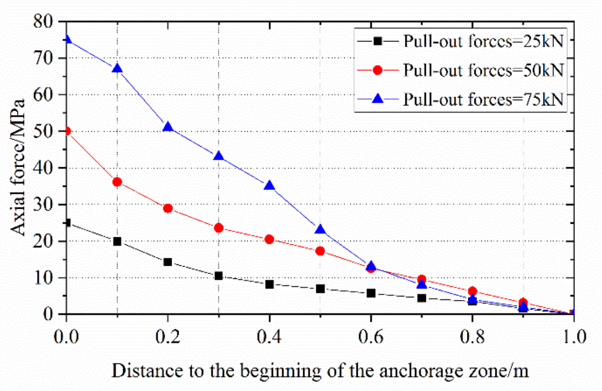

**Fig 10. Distributions of shear stress in the anchorage zone under different pull-out forces at the same anchorage length.** (a) 0.5 m; (b) 1.0 m.

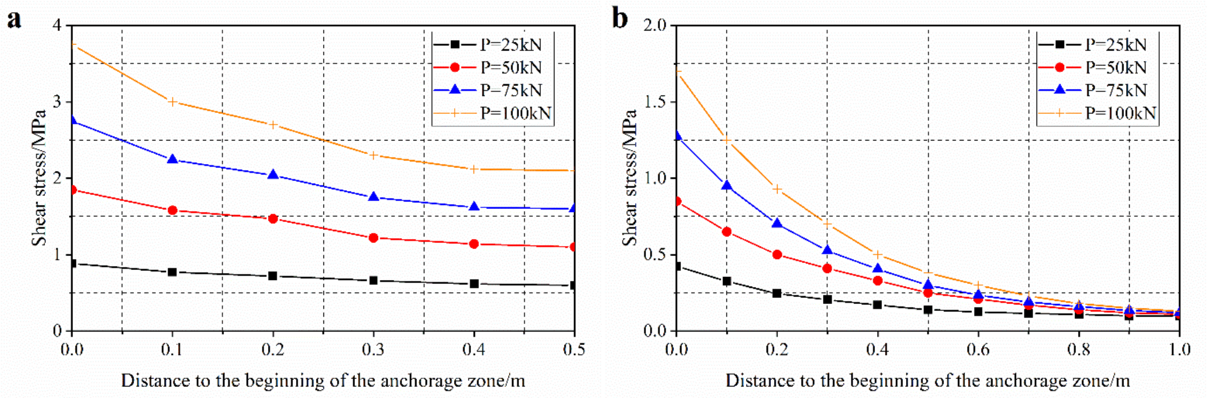

**Fig 11. Relationships between peak, and incremental, axial force in the anchorage zone with anchorage length.**

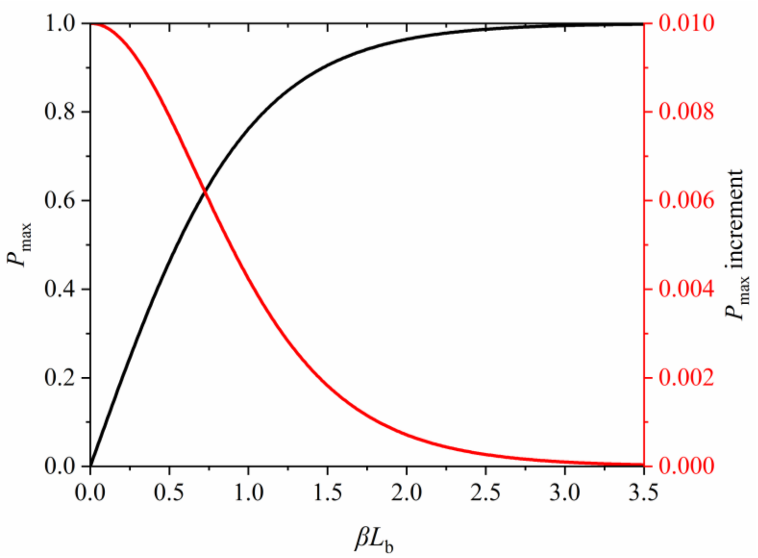

**Fig 12. Relationships between peak, and incremental, axial forces in the anchorage zone with anchorage length.**

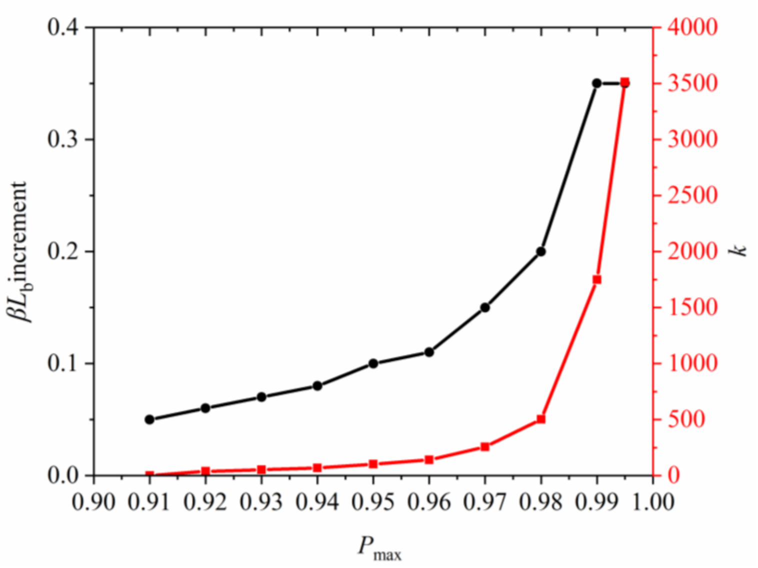

Figure data

Click here to access/download
**Supporting Information**
Figure data.xlsx

Click here to access/download
**Supporting Information**
Language Revision Certificate.pdf
