# Supplementary material for: Load transfer mechanism and critical length of anchorage zone for anchor bolt
Source: PLoS One. 2020 Jan 17;15(1):e0227539. doi: 10.1371/journal.pone.0227539 (PMC6968847; doi:10.1371/journal.pone.0227539)
Supplement: Attachment — Submitted filename: Response to the Reviewers.doc [file pone.0227539.s006.doc]

Dear Editor:

Thank you very much for your letter on 6 September, 2019, regarding the comments and suggestions made on our paper entitled “Load transfer mechanism and critical length of anchorage zone for anchor bolt” (ID: PONE-D-19-21977). The comments are very valuable and very helpful to us when revising and improving our manuscript, as well as the important guiding significance to our researches. We have studied the comments carefully and have made the required revisions.

Enclosed are a clean copy of our revised manuscript (see ‘Manuscript-Revised’), a marked-up copy of our manuscript that highlights changes made to the original version (see ‘Revised Manuscript with Track Changes’ and ‘Manuscript-Revised _HighlightedCopy’), and a detailed response letter to the editors and reviewers explaining the changes we have made (see ‘Response to the Reviewers’).

The main revisions to the manuscript and our response to you and the reviewers’ comments are as follows:

Journal Requirement:

1. Please include in your Methods section the vendor details for all equipment and materials used, in order to enhance reproducibility.

Response: Thank the Editor for the kind recommendation. The LW-1000 horizontal tensile test machine is provided by Hangzhou Yingmin Technology Co., Ltd(Zhejiang China). The left-handed threaded steel anchor bolts and the resin cartridge are provided by Xuzhou Baoding Support Technology Co., Ltd(Jiangsu China). The strain gauges and TS3890 static resistance strain gauges are provided by Jiangsu Donghua Testing Technology Co., Ltd(Jiangsu China).

2. We note you have included a table to which you do not refer in the text of your manuscript. Please ensure that you refer to Table 2 in your text; if accepted, production will need this reference to link the reader to the Table.

Response: We are very sorry for our negligence and thanks for the reviewer’s kind suggestion. We have described and referenced Table 2 in the text. The revised details can be found in Line 130-132, Page 5(Revised Manuscript).

Reviewer Comment:

1. Is the manuscript technically sound, and do the data support the conclusions?

Response: Thanks for the reviewer. The conclusions of this paper are obtained by theoretical analysis, laboratory test and numerical simulation. The data of this paper are all real data. The result of data analysis is promoted to the theoretical level, so as to reach the final conclusion, which is in line with the logic thinking and methods commonly used in scientific research. Moreover, the operation process of the laboratory test in this paper conforms to the test specifications, so the data obtained is reliable enough to support the conclusion of this paper.

2. Has the statistical analysis been performed appropriately and rigorously?

Response: Thanks for the reviewer.All the data in this paper are real data. The analysis of the data was carried out according to the corresponding scientific norms without any falsification. The conclusions of this paper are based on the real data and scientific and technological standards, and the conclusions are credible. Therefore, the author thinks that the statistical analysis is appropriate and rigorous.

3. Have the authors made all data underlying the findings in their manuscript fully available?

Response: Thanks for the reviewer’s kind suggestion. The curves in this paper are all based on the obtained data, which can reflect the data and its changing trend. If the data supporting all graphs is added to the paper, it will not only duplicate the contents of graphs, but also lead to the verbose and complex structure of the paper. Therefore, all data are provided separately. Please refer to attached files for details.

4. Is the manuscript presented in an intelligible fashion and written in standard English?

Response: Thank the Reviewer for the kind recommendation. The English throughout has been revised by the native English-speaking professors using track change in MS Word. ‘Language Revision with Track Change.doc’ and ‘Language Revision Certificate.pdf’ issued by the service, are all uploaded for your references.

5. ①The laboratory testing campaign was performed. At least some summary of the number of conducted tests (with the description of every group of tested anchors regarding lengths) should be presented. Otherwise the support of the conclusions by the experiments can´t be evaluated properly.

Response: Thanks for the reviewer’s kind suggestion. In this paper, the mechanical properties of the anchorage body of the anchor under different anchorage lengths (500mm、1000mm and 1500mm) and pull-out forces (25kN、50kN、75kN、100kN) are tested. Each scheme was tested twice, so a total of 14 tests were carried out, and then the test data were analyzed and selected. We add the above summary to the paper. The revised details can be found in Line 149-150 and Page 6.

②The analytical model which was used is not applicable without the knowledge of shear stress and shear displacement values related to the specific anchor. Stress-strain diagram of tested anchors (Or at least the values of stress and strain which are essential for the use of mentioned model) is worth to include in article. It is in line with the demand of availability of the data underlying the findings.

Response: Thanks for the reviewer’s kind suggestion. This paper focuses on the mechanical characteristics of the anchorage section in the elastic stage. According to the previous studies (reference 29, 30, etc.), the mechanical model adopted in this paper is the most consistent with the shear stress displacement relationship of the anchorage interface in the elastic stage, and the experimental research in the following paper also verifies the correctness of the model. At the same time, according to the suggestions put forward by the reviewer, the relationship diagram between pull-out force and displacement and failure form diagram of the anchor during the test process were added, which provides more evidence for the credibility of the data on which the research results are based. The revised details can be found in Line 153-160, page 4.

③The more attention should be paid to the logical organizing structure of text. There are some results presented in the paragraph 3.3 (Results). Then the methodology of numerical modelling is mentioned (Methods). From this point in the manuscript it can starts to be complicated to the reader to distinguish which discussed results are related to the laboratory experiment and which to the numerical simulation. It would be also good to improve the captions of graphs in this way.

Response: Thanks for the Reviewer. According to the reviewer's suggestion, the author has reorganized the article structure of the third part in the form of research method-research scheme-research results. Please see the new manuscript for details. The revised details can be found in Line 116-117,129-143,161-162,187,205-206,213, page 5-8.

We would like to thank the Reviewers for the kind comments and appreciate the Editor’s and Reviewers’ diligence. We trust that the revisions will meet with your approval.

Once again, thank you very much for your time in this matter.

Yours sincerely,

Xingliang Xu, Suchuan Tian
