# Supplementary material for: Load transfer mechanism and critical length of anchorage zone for anchor bolt
Source: PLoS One. 2020 Jan 17;15(1):e0227539. doi: 10.1371/journal.pone.0227539 (PMC6968847; doi:10.1371/journal.pone.0227539)
Supplement: Attachment — Submitted filename: Response to the Reviewers.doc [file pone.0227539.s007.doc]

Dear Editor:

Thank you very much for your letter on 15 November, 2019, regarding the comments and suggestions made on our paper entitled “Load transfer mechanism and critical length of anchorage zone for anchor bolt” (ID: PONE-D-19-21977 R1). The comments are very valuable and very helpful to us when revising and improving our manuscript, as well as the important guiding significance to our researches. We have studied the comments carefully and have made the required revisions.

Enclosed are a clean copy of our revised manuscript (see ‘Revised Manuscript’), a copy of revised manuscript with track changes (see ‘Revised Manuscript with Track Changes’), and a detailed response letter to the editors and reviewers explaining the changes we have made (see ‘Response to Reviewers’).

The main revisions to the manuscript and our response to the reviewers’ comments are as follows:

1. The term “different” used in the recently added lines 134-136 need to be explained. I cite: …” … deformation characteristics of different interface of anchorage are different”.” … model was adjusted by setting different contact mechanical parameters”. The values of parameters mentioned in these sentences should be added into the manuscript.

Response: Thanks for the reviewer for the good comment. I have added two interface parameters mentioned by reviewers in the new manuscript, namely, "In the anchor bolt–anchoring agent interface, the ultimate shear stress and shear stiffness are 8 MPa and 500 MPa/m. In the anchoring agent–borehole wall interface, the ultimate shear stress and shear stiffness are 4.5 MPa and 300 MPa/m." The revised details can be found in Line 135-137 and Page 5.

2. Some extra details about the sensors used for laboratory testing has been introduced by the authors. However, even with this new information, I am still asking one question to the authors: “How were the strain gauges (e. g. 15 gauges in case of Lb = 1500 mm) connected to the datalogger? If a real photo of rod with installed gauges (and/or detail of installation of gauge on the threaded bar) exists, I encourage the authors to publish it, in order to enhance reproducibility of their work. (It can be added, for example, to the fig. 5).

Response: Thanks for the Reviewer. The wire is connected with one end of the strain gauge. The strain gauge is pasted on the corresponding position of the anchor rod with glue. The wire is led out from the near end of the steel pipe and connected with the data collecting instrument. Place the bolt with strain gauge in the center of the steel pipe, and quickly inject the mixed anchoring agent. The test can be carried out after the anchoring agent solidifies. Due to the author's negligence, some test scenes were not photographed during the test. In order to make up for the author's negligence and provide the following scholars with experimental reference, the author has made a schematic diagram of the installation structure of anchor bolt and strain gauge. The revised details can be found in page 56 in “Revised Figures.doc”.

We would like to thank the Reviewers for the kind comments and appreciate the Editor’s and Reviewers’ diligence. We trust that the revisions will meet with your approval.

Once again, thank you very much for your time in this matter.

Thank you and best regards.

Yours sincerely,

Xingliang Xu, Suchuan Tian

tiansc@cumt.edu.cn
